# Long-Term Outcomes Associated with Traumatic Brain Injury in Childhood and Adolescence: A Nationwide Swedish Cohort Study of a Wide Range of Medical and Social Outcomes

**DOI:** 10.1371/journal.pmed.1002103

**Published:** 2016-08-23

**Authors:** Amir Sariaslan, David J. Sharp, Brian M. D’Onofrio, Henrik Larsson, Seena Fazel

**Affiliations:** 1 Department of Psychiatry, University of Oxford, Warneford Hospital, Oxford, United Kingdom; 2 Computational, Cognitive and Clinical Neuroimaging Laboratory, Imperial College, London, United Kingdom; 3 Department of Psychological and Brain Sciences, Indiana University, Bloomington, Indiana, United States of America; 4 Department of Medical Epidemiology and Biostatistics, Karolinska Institutet, Stockholm, Sweden; 5 School of Medical Sciences, Örebro University, Örebro, Sweden; Western Sydney University, AUSTRALIA

## Abstract

**Background:**

Traumatic brain injury (TBI) is the leading cause of disability and mortality in children and young adults worldwide. It remains unclear, however, how TBI in childhood and adolescence is associated with adult mortality, psychiatric morbidity, and social outcomes.

**Methods and Findings:**

In a Swedish birth cohort between 1973 and 1985 of 1,143,470 individuals, we identified all those who had sustained at least one TBI (*n* = 104,290 or 9.1%) up to age 25 y and their unaffected siblings (*n* = 68,268) using patient registers. We subsequently assessed these individuals for the following outcomes using multiple national registries: disability pension, specialist diagnoses of psychiatric disorders and psychiatric inpatient hospitalisation, premature mortality (before age 41 y), low educational attainment (not having achieved secondary school qualifications), and receiving means-tested welfare benefits. We used logistic and Cox regression models to quantify the association between TBI and specified adverse outcomes on the individual level. We further estimated population attributable fractions (PAF) for each outcome measure. We also compared differentially exposed siblings to account for unobserved genetic and environmental confounding. In addition to relative risk estimates, we examined absolute risks by calculating prevalence and Kaplan-Meier estimates. In complementary analyses, we tested whether the findings were moderated by injury severity, recurrence, and age at first injury (ages 0–4, 5–9, 6–10, 15–19, and 20–24 y).

TBI exposure was associated with elevated risks of impaired adult functioning across all outcome measures. After a median follow-up period of 8 y from age 26 y, we found that TBI contributed to absolute risks of over 10% for specialist diagnoses of psychiatric disorders and low educational attainment, approximately 5% for disability pension, and 2% for premature mortality. The highest relative risks, adjusted for sex, birth year, and birth order, were found for psychiatric inpatient hospitalisation (adjusted relative risk [aRR] = 2.0; 95% CI: 1.9–2.0; 6,632 versus 37,095 events), disability pension (aRR = 1.8; 95% CI: 1.7–1.8; 4,691 versus 29,778 events), and premature mortality (aRR = 1.7; 95% CI: 1.6–1.9; 799 versus 4,695 events). These risks were only marginally attenuated when the comparisons were made with their unaffected siblings, which implies that the effects of TBI were consistent with a causal inference. A dose-response relationship was observed with injury severity. Injury recurrence was also associated with higher risks—in particular, for disability pension we found that recurrent TBI was associated with a 3-fold risk increase (aRR = 2.6; 95% CI: 2.4–2.8) compared to a single-episode TBI. Higher risks for all outcomes were observed for those who had sustained their first injury at an older age (ages 20–24 y) with more than 25% increase in relative risk across all outcomes compared to the youngest age group (ages 0–4 y). On the population level, TBI explained between 2%–6% of the variance in the examined outcomes.

Using hospital data underestimates milder forms of TBI, but such misclassification bias suggests that the reported estimates are likely conservative. The sibling-comparison design accounts for unmeasured familial confounders shared by siblings, including half of their genes. Thus, residual genetic confounding remains a possibility but will unlikely alter our main findings, as associations were only marginally attenuated within families.

**Conclusions:**

Given our findings, which indicate potentially causal effects between TBI exposure in childhood and later impairments across a range of health and social outcomes, age-sensitive clinical guidelines should be considered and preventive strategies should be targeted at children and adolescents.

## Abstract 2

In a population-wide observational cohort, Seena Fazel and colleagues use a sibling-matched design to examine the burden of long-term outcomes associated with traumatic brain injury.

## Author Summary

**Why Was This Study Done?:**

- Traumatic brain injury (TBI) constitutes the leading cause of morbidity and mortality in individuals under the age of 45 y globally.
- Research on the long-term effects of TBI is limited to more severe injuries and medical outcomes.
- There is uncertainty whether children and adolescents experiencing milder forms of TBI may have significant medical and social problems in adulthood.

**What Did the Researchers Do and Find?:**

- We used national registers in Sweden covering 1.1 million individuals born between 1973–1985
- In the 9.1% who sustained at least one TBI before the age of 25 y, we examined later risk of six medical and social outcomes.
- We compared TBI patients with their unaffected siblings in order to account for the possibility that the risk for these outcomes runs in families.
- We found TBI consistently predicted later risk of premature mortality, psychiatric inpatient admission, psychiatric outpatient visits, disability pension, welfare recipiency, and low educational attainment in the sibling-comparison analyses, and the effects were stronger for those with greater injury severity, recurrence, and older age at first injury.

**What Do These Findings Mean?:**

- Consideration needs to be given to review the cognitive, psychiatric, and social development all children and adolescents who sustain head injuries.
- Guidelines should consider age-specific recommendations for follow-up.
- The public health benefits of preventing TBIs should include social outcomes.

## Introduction

The World Health Organization ranks traumatic brain injury (TBI) as the leading cause of both disability and mortality in individuals below the age of 45 y [1]. Conservative estimates from the United States indicate that TBI accounts for an annual combined average of approximately 848,000 hospital admissions and emergency room visits in individuals under the age of 25 y, the majority of which classified as concussions or mild TBI [2]. In England and Wales, it is estimated that between 462,200 and 700,000 children under 15 y of age attend emergency departments annually with head injury [3]. Similar findings have been reported in other high-income countries [4–6].

Epidemiological studies have consistently found higher rates of adverse outcomes in adults who have sustained a TBI when compared to the general population [7–16]. However, there is a lack of large-scale studies that have examined the potential long-term impact of TBI exposure during childhood and adolescence. Importantly, there is a gap in the literature identifying how injury characteristics (e.g., severity, recurrence, and developmental timing) might moderate such associations. Previous work suggests that TBI in children contributes to poorer measures of emotion perception and social interaction [17,18], employment, and quality of life [19,20]. Nevertheless, uncertainty remains as to how early TBI exposure is related to a broader set of specific medical and social functioning outcomes in adulthood [10,21,22], such as premature mortality, disability pension, and educational attainment. Some of this uncertainty is attributed to small and selected clinical samples, poor adjustments for confounding factors, and non-specific outcomes that measure global disabilities.

Therefore, we have used population-based registers of the entire Swedish population born between 1973 and 1982 to examine how exposure to a TBI from birth up to age 25 y is associated with functioning in adulthood. These datasets have allowed us to examine the extent to which injury severity and recurrent injuries predict a range of long-term outcomes. Injuries to the brain during developmentally sensitive periods may be particularly problematic [23,24]. We have also separately considered the associations between TBI experienced during different developmental stages during childhood and adolescence and the adverse functioning outcomes in adulthood. A unique feature of this study is the use of the sibling-comparison design [25]. This has been adopted in previous Swedish studies [8,16] and allows one to account for unobserved familial (e.g., genetic and environmental) confounders. This is important because previous research has demonstrated that indicators of medical and social functioning are considerably heritable [26–28]. By comparing differentially exposed siblings, we were able to account for an aggregate of their shared genetic and environmental risks, which allowed us to estimate more accurate estimates of the consequences of early TBI exposure.

## Methods

### Swedish Nationwide Registers

This study was approved by the Regional Research Ethics Committee in Stockholm (RRECiS; 2013/5:8). Statistics Sweden, an independent governmental agency, maintains a number of nationwide longitudinal registries with routinely gathered governmental agency data. A ten-digit civic registration number assigned to all Swedish residents enables them to merge all of these registers. Following the approval from the RRECiS, we were given access to anonymised data.

The Total Population/Multi-Generation Register (TPR/MGR; 1932–2014) linked all Swedish residents to their biological parents, allowing us to identify all biological full siblings. Emigration and mortality dates were derived from the Migration and the Causes of Death Registers (1973–2014), respectively. The National Patient Register provided data on inpatient care (based on International Classification of Diseases eighth, ninth, and tenth editions [ICD-8, ICD-9 and ICD-10] codes; 1973–2013) and outpatient care (ICD-10; 2001–2013). Parental criminal convictions were gathered from the National Crime Register, which includes all criminal convictions in lower general court in Sweden (1973–2013). Annual sociodemographic data were derived from the following census registers covering all Swedish residents who were alive at the end of each year: the Education Register (1985–2012), the Population and Housing Register (1985), and the Integrated Database for Labour Market Research (LISA; 1990–2012).

### Sample

Our initial sample included all Swedish children born between 1973 and 1985 (*n* = 1,333,919). In the following order, we excluded those who could not be linked to both of their biological parents (*n* = 63,018), had died (*n* = 16,806) or had migrated (*n* = 95,327) before the age of 26 y, as well as those who lacked data on parental sociodemographic factors (*n* = 3,427) and adulthood outcome measures (*n* = 11,871). The final sample therefore consisted of 1,143,470 children with complete data, of which 683,860 had at least one biological full-sibling. We found no evidence of non-random selection effects in the excluded sample (S1 Text).

### Measures

#### Definition of TBI

We defined an individual who had sought treatment for at least one episode of a concussion (mild TBI) or a moderate-to-severe TBI before 25 y of age as a TBI patient. The patients were diagnosed in specialist care according to ICD-criteria (see S1 Table). Mild TBI diagnoses recorded in the Swedish National Patient Register have demonstrated excellent validity (positive predictive value = 100%; negative predictive value = 99.8%) [29]. Similar results have also been found for unintentional injuries [30]. To examine the effects of injury severity, we also generated separate measures for mild TBI and moderate to severe TBI. Individuals who had sustained a mild and a moderate-to-severe TBI were only included in the omnibus TBI measure. We defined recurrence as two or more episodes of TBI. To avoid bias resulting from duplicate records of the same episode in the registries, we excluded episodes that had occurred within 15 d of one another.

We examined developmentally sensitive periods by stratifying the group of TBI patients according to their age at first injury using the same age categories as the US Centers for Disease Control and Prevention [2], namely 0–4 y, 5–9 y, 10–14 y, 15–19 y, and 20–24 y. To investigate whether our findings were specific to TBI or to a general proneness to injury, we additionally examined a group of individuals who had been through at least one episode of fall-related injury but who had not been diagnosed with a TBI in a sensitivity test.

#### Outcomes

Six outcome variables that assessed specific dimensions of both medical and social functioning were examined. The baseline of the study started during the year that the individuals turned 26 y of age and we followed them in the registers until they experienced the outcome, migrated, died, or exited the study (e.g., the end of 2012–2014 depending on the outcome of interest). “Disability pension” measured whether the individual was in receipt of disability pension (covering up to 65% of lost wages) due to permanent work incapacity [31]. “Psychiatric visit” or “psychiatric hospitalisation” measured whether the individual had visited an outpatient clinic or been admitted to inpatient care for any psychiatric diagnosis. “Premature mortality” indicated whether the individual had died before a maximum age of 41 y. “Low educational level” indicated that the highest achieved attainment did not meet secondary school qualifications as defined by the SUN2000 classification system [32], at the baseline of the follow-up (age 26 y). “Welfare recipiency” indicated whether the individual had received means-tested welfare benefits [33].

#### Covariates

The TPR provided information on sex, birth year (continuous), and birth order (first, second, third, and fourth or higher). Parental lifetime history of psychiatric morbidity and criminality were measured as binary variables, indicating whether either biological parent of the individuals had been diagnosed for any psychiatric disorder or convicted of a criminal offence. We measured parental sociodemographic factors in 1985. If the information was missing, we used the earliest available information up until 2012. “Family income” was defined as the disposable income, which includes both earnings and benefits, averaged across both biological parents. “Low parental education level” measured whether neither biological parent had achieved secondary school qualifications. “Maternal single status” indicated that the mother was not married.

### Analytical Approach

We initially generated descriptive statistics to examine how sociodemographic characteristics were distributed across TBI exposure groups with varying degrees of injury severity.

We then estimated the magnitude of the associations between TBI and the functional outcomes of interest by fitting a series of logistic and Cox regression models that accommodated the binary and right-censored outcome distributions. A total of 42,132 individuals had emigrated and 5,494 individuals had died during the follow-up period and were right-censored. Time at risk was, therefore, explicitly accounted for in the Cox regression model. We will henceforth refer to the effect measures (e.g., odds and hazard ratios) as risk ratios. We gradually accounted for a number of measured covariates by fitting two separate models to the full sample (*n* = 1,143,470). In these analyses, we assumed that all individuals were unrelated to one another. In Model I, we only adjusted for sex, birth year, and birth order. Model II additionally adjusted for individual and parental educational level, parental lifetime history of psychiatric morbidity and criminality, family income, as well as maternal single status. In Model III, we further tested for the contribution of unobserved familial confounders by fitting a sibling-comparison model to the sibling subsample (*n* = 683,860) using fixed-effects estimators [34] of the statistical models described above, which stratified the estimates across clusters of biological parents.

The rationale for Model III was to compare risks of poor adulthood functioning in differentially exposed full-siblings (i.e., one sibling has sustained a TBI and the other one has not; *n* = 108,086) and thereby indirectly control for an aggregate of factors that make the siblings similar to one another (e.g., 50% of their co-segregating genes and their shared childhood environments). This implies that Model III indirectly accounted for all of the measured family-wide covariates (e.g., family income and maternal single status) in Model II, as they are constant between siblings. Under these assumptions, Model III isolated the residual effect of the association of interest, which is consistent with a causal inference [25]. The magnitude of familial confounding is measured as the relative attenuation of the within-family estimate compared to the unrelated population estimate [35]. We followed the STROBE guidelines for cohort studies (see S1 STROBE Checklist for details).

#### Sensitivity analyses

We re-fitted Models I–III to non-TBI fall-related injury to examine whether the findings were specific to TBI or to a general proneness to injury. To examine whether recurrent TBIs was a stronger risk factor than a single-episode TBI, we re-specified Models I–III so that we compared individuals who had sustained recurrent TBIs with those who had sustained a single TBI, rather than those who had not sustained any TBIs. By definition, this implies that Model III examined the relative risk differences between siblings that were differentially exposed to either recurrent TBIs and single TBI. We explored potential moderation effects by age at first TBI by re-fitting Models I–III to the five age category measures. To maximise statistical power in these analyses for premature mortality, we defined the baseline of the follow-up at the end of each exposure period (e.g., at ages 4, 9, 14, 19, and 24 y).

We were asked to conduct the following complementary sensitivity analyses during the peer review process: We tested whether our findings could be explained by any comorbid psychiatric and/or neurological conditions that had occurred at any time throughout the exposure period, up to the age of 25 y, by re-running Models I–III on a subsample that excluded individuals who met such criteria (*n* = 83,897). To explore time trends, we tested whether the birth year moderated the effects of TBI on the outcomes by adding an interaction term to Model III. We also considered whether the correlated nature of our outcome measures (correlation table presented in S2 Table) could impact on the presented findings by comparing the Model I estimates to equivalent estimates derived from a multivariate structural equation model (SEM), where all of the associations were tested jointly. In the SEM, we specified the right-censored outcome variables to be treated as Poisson distributed counts with offset variables, which allowed us to explicitly take time at risk into account. We also calculated population attributable fractions (PAF), or the relative share of the outcomes that could be attributed to TBI. Similar to the individual-level analyses described above, we gradually accounted for confounding factors using the Models I–III specifications.

We used Stata 14 MP [36] to fit the logistic regression (logit), conditional logistic regression (xtlogit, fe), Cox regression (stcox), stratified Cox regression (stcox, strata()), and multivariate SEM (gsem) models, as well as to estimate PAFs (punafcc) for Models I and II. We used the AF 0.1.1 package [37] in R 3.2.2 [38] to estimate PAFs for Model III.

## Results

We identified 104,290 (9.1%) individuals that had sustained a TBI before age 25 y (Table 1). The overwhelming majority of these persons had suffered from mild TBIs (*n* = 80,676; 77.4%) and one in eight (*n* = 12,680; 12.2%) had experienced recurrent injuries. People with head injuries were, on average, more likely to be male and to have grown up in households characterised by a range of adverse psychosocial indicators (Table 1). Individuals who had sustained recurrent TBIs were, on average, approximately 2 y younger at their first injury than those who had sustained a single TBI (12.0 versus 13.8 y). Those who had sustained mild TBIs also tended to be younger at the time of their first injury than those who had sustained moderate to severe TBIs (12.2 versus 19.2 y).

**Table 1:** Sociodemographic characteristics for the sample (*n* = 1,143,470).

	TBI			Mild TBI			Moderate to severe TBI		
	Never (*n* = 1,039,180)	Once (*n* = 91,610)	Recurrent (*n* = 12,680)	Never (*n* = 1,062,794)	Once (*n* = 73,330)	Recurrent (*n* = 7,346)	Never (*n* = 1,119,856)	Once (*n* = 21,187)	Recurrent (*n* = 2,427)
Male gender, n (%)	523,610 (50.4%)	58,027 (63.4%)	9,154 (72.2%)	541,588 (51.0%)	44,372 (60.5%)	4,831 (65.8%)	572,813 (51.2%)	15,993 (75.5%)	1,993 (81.8%)
Low parental education level, n (%)	178,877 (17.2%)	16,696 (18.5%)	2,563 (20.2%)	183,003 (17.2%)	13,898 (19.0%)	1,508 (20.5%)	194,283 (17.4%)	3,653 (17.2%)	473 (19.5%)
Family income in the lowest decile, n (%)	100,771 (9.9%)	9,797 (11.0%)	1,531 (12.4%)	103,368 (10.0%)	7,840 (11.0%)	891 (12.5%)	109,502 (10.0%)	2,309 (11.2%)	288 (12.3%)
Maternal single status, n (%)	301,251 (29.0%)	30,765 (33.6%)	4,807 (37.9%)	309,947 (29.2%)	24,163 (33.0%)	2,713 (36.9%)	328,127 (29.3%)	7,764 (36.7%)	932 (38.4%)
Lifetime parental psychiatric morbidity, n (%)	258,546 (24.9%)	26,461 (28.9%)	4,187 (33.0%)	265,314 (25.0%)	21,398 (29.2%)	2,482 (33.8%)	282,426 (25.2%)	6,027 (28.5%)	741 (30.5%)
Lifetime parental criminal history, n (%)	412,093 (39.7%)	42,365 (46.2%)	6,722 (53.0%)	423,820 (39.9%)	33,551 (45.8%)	3,809 (51.9%)	449,453 (40.1%)	10,393 (49.1%)	1,334 (55.0%)
Mean age at first diagnosis, years (s.e.m.)	N/A	13.8 (0.02)	12.0 (0.06)	N/A	12.2 (0.02)	9.5 (0.07)	N/A	19.2 (0.04)	18.4 (0.10)

Notes: N/A, not applicable; s.e.m., Standard error of the mean

**Fig 1:**
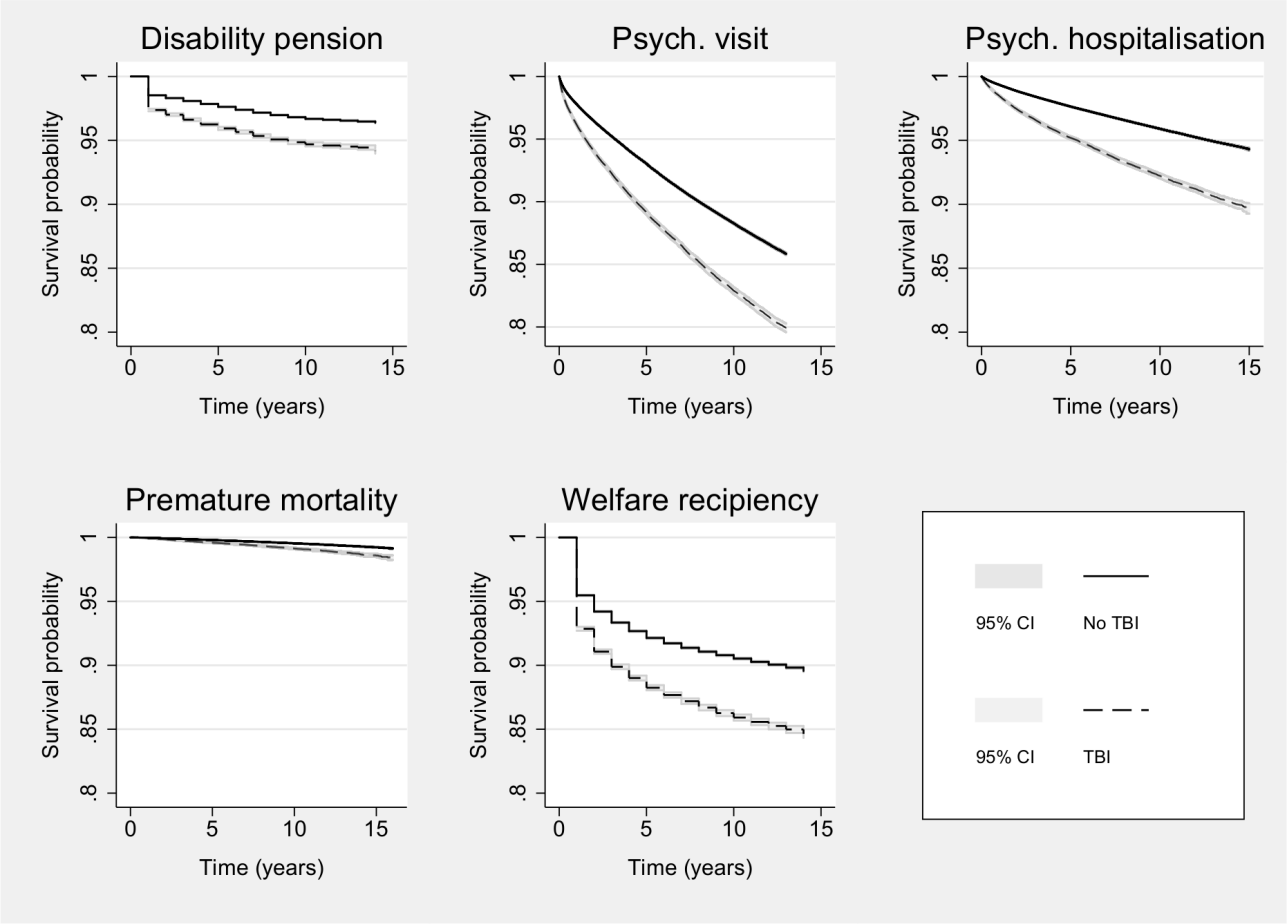
Kaplan-Meier survival curves for all right-censored outcomes across TBI exposure before age 25 y.

TBI was associated with elevated risk of impaired adult functioning across all outcome measures (Fig 1; Table 1; complete list of absolute risks in S3 Table). Sustaining a TBI contributed to absolute risks of over 10% for specialist diagnoses of psychiatric disorders, low educational attainment, and welfare recipiency, and approximately 6% for disability pension. In terms of relative risks (Table 2), we found that the risk of disability pension increased by 76% (RR = 1.76; 95% CI: 1.71–1.82), which reduced to 49% (RR = 1.49; 1.38–1.60) when the comparison was made with unaffected siblings. The strongest association was found for psychiatric inpatient hospitalisation, for which the risk was doubled (RR = 1.95; 1.90–2.10). When compared to their unaffected siblings, TBI patients continued to be nearly 60% (RR = 1.57; 95% CI: 1.47–1.67) more likely to be hospitalised for any psychiatric disorder. Social functioning impairments were also more common in those who had sustained a TBI, being 60% more likely than unrelated controls (RR = 1.58; 1.55–1.61) and 28% more likely than their unaffected siblings (RR = 1.28; 1.23–1.33) to have attained a low level of education in adulthood. Similar results were observed for receiving welfare benefits. These findings could not be attributed to comorbid psychiatric and/or neurological conditions throughout the exposure period (S4 Table). Multivariate analyses taking the correlation between the outcomes into account were also commensurate with these findings (Table 3). Furthermore, we did not find any statistically significant (*p* < 0.05) moderation effects by birth year, thus suggesting that the effects of TBI on the outcomes did not change materially over time.

**Table 2:** Relative risks (RR) and corresponding 95% confidence intervals (CIs) for the associations between TBI before age 25 y and poor functioning in adulthood.

	Model I	Model II	Model III
	RR [95% CI]	RR [95% CI]	RR [95% CI]
Disability pension	1.76 [1.71; 1.82]	1.47 [1.43; 1.52]	1.49 [1.38; 1.60]
Psychiatric visit	1.52 [1.50; 1.55]	1.37 [1.35; 1.40]	1.31 [1.26; 1.37]
Psychiatric hospitalisation	1.95 [1.90; 2.00]	1.69 [1.64; 1.73]	1.57 [1.47; 1.67]
Premature mortality	1.73 [1.60; 1.86]	1.50 [1.39; 1.62]	1.40 [1.16; 1.68]
Low education	1.58 [1.55; 1.61]	1.43 [1.41; 1.46]	1.28 [1.23; 1.33]
Welfare recipiency	1.55 [1.52; 1.58]	1.30 [1.27; 1.32]	1.19 [1.14; 1.23]

Notes: Model I: Full sample, adjusted for sex, birth order, and birth year. Model II: Additional adjustments for individual and parental highest achieved education levels, parental income, parental lifetime criminal and psychiatric histories, and maternal single status. Model III: Within-family estimates that are additionally adjusted for individual educational attainment at age 26 y.

**Table 3:** Relative risks (RR) and corresponding 95% confidence intervals (CIs) for the associations between TBI before age 25 y and poor functioning in adulthood, assessed in univariate and multivariate models.

	Standard univariate models (Reference)	Multivariate structural equation model
	RR [95% CI]	RR [95% CI]
Disability pension	1.76 [1.71; 1.82]	1.77 [1.71; 1.82]
Psychiatric visit	1.52 [1.50; 1.55]	1.53 [1.50; 1.56]
Psychiatric hospitalisation	1.95 [1.90; 2.00]	1.95 [1.90; 2.00]
Premature mortality	1.73 [1.60; 1.86]	1.72 [1.60; 1.86]
Low education	1.58 [1.55; 1.61]	1.58 [1.55; 1.61]
Welfare recipiency	1.55 [1.52; 1.58]	1.56 [1.53; 1.59]

Notes: Both models are adjusted for sex, birth order, and birth year.

We found that the crude population contributions of TBI explained approximately 4%–7% of the differences in the outcome measures (Table 4; Model I). These estimates were partially attenuated once familial confounders were accounted for (Model III) but remained statistically significant, explaining between 2%–6% of the population differences in the outcomes. The strongest population attributable risks were found for the severe outcomes, including psychiatric inpatient hospitalisation (PAF = 5.5%; 4.9%–6.1%), premature mortality (PAF = 4.7%; 2.9%–6.5%), and disability pension (PAF = 4.6%; 3.8%–5.3%).

**Table 4:** Population-attributable fractions, expressed as percentages, for TBI before age 25 y on poor functioning in adulthood.

	Model I	Model II	Model III
	PAF [95% CI]	PAF [95% CI]	PAF [95% CI]
Disability pension	5.9% [5.7%; 6.2%]	4,5% [4.2%; 4.8%]	4.6% [3.8%; 5.3%]
Psychiatric visit	4.4% [4.2%; 4.5%]	3.4% [3.3%; 3.6%]	3.1% [2.7%; 3.4%]
Psychiatric hospitalisation	7.4% [7.3%; 7.7%]	6.4% [6.1%; 6.6%]	5.5% [4.9%; 6.1%]
Premature mortality	6.1% [5.5%; 6.8%]	5.0% [4.2%; 5.7%]	4.7% [2.9%; 6.5%]
Low education	5.0% [4.8%; 5.1%]	4.1% [3.9%; 4.3%]	2.8% [2.4%; 3.3%]
Welfare recipiency	4.7% [4.5%; 4.8%]	3.2% [3.0%; 3.4%]	2.4% [1.9%; 2.9%]

Notes: Model I: Full sample, adjusted for sex, birth order, and birth year; Model II: Additional adjustments for individual and parental highest achieved education levels, parental income, parental lifetime criminal and psychiatric histories, and maternal single status; Model III: Within-family estimates that are additionally adjusted for individual educational attainment at age 26 y.

Individuals that had sustained non-TBI fall injuries were generally less likely than those who had sustained a TBI to have poor adulthood outcomes (Table 5). The differences were most noticeable for severe outcomes, such as disability pension, for which the risk increase for non-TBI patients compared to their unaffected siblings (RR = 1.01; 0.91–1.12) was less than the equivalent risk estimate for TBI patients (RR = 1.49; 1.38–1.60). We also found similar results for premature mortality.

**Table 5:** Relative risks (RR) and corresponding 95% confidence intervals (CIs) for the associations between non-TBI fall injuries, mild TBI and moderate to severe TBI up to age 25 y on adulthood poor functioning.

	Model I	Model II	Model III
	RR [95% CI]	RR [95% CI]	RR [95% CI]
**Non-TBI fall injury**			
Disability pension	1.14 [1.09; 1.20]	1.09 [1.03; 1.14]	1.01 [0.91; 1.12]
Psychiatric visit	1.15 [1.12; 1.18]	1.11 [1.08; 1.14]	1.14 [1.08; 1.21]
Psychiatric hospitalisation	1.28 [1.23; 1.34]	1.23 [1.18; 1.28]	1.26 [1.15; 1.38]
Premature mortality	1.21 [1.06; 1.39]	1.17 [1.02; 1.33]	1.14 [0.86; 1.50]
Low education	1.14 [1.11; 1.17]	1.11 [1.08; 1.14]	1.05 [0.99; 1.11]
Welfare recipiency	1.09 [1.06; 1.12]	1.04 [1.01; 1.07]	1.04 [0.98; 1.11]
**Mild TBI**			
Disability pension	1.66 [1.61; 1.72]	1.40 [1.35; 1.45]	1.36 [1.25; 1.47]
Psychiatric visit	1.50 [1.47; 1.53]	1.36 [1.33; 1.38]	1.31 [1.25; 1.36]
Psychiatric hospitalisation	1.91 [1.86; 1.97]	1.67 [1.62; 1.71]	1.52 [1.42; 1.63]
Premature mortality	1.60 [1.47; 1.75]	1.40 [1.29; 1.53]	1.26 [1.02; 1.55]
Low education	1.55 [1.52; 1.58]	1.41 [1.37; 1.44]	1.25 [1.19; 1.31]
Welfare recipiency	1.52 [1.49; 1.56]	1.28 [1.26; 1.32]	1.18 [1.13; 1.23]
**Moderate to severe TBI**			
Disability pension	2.19 [2.06; 2.32]	1.78 [1.67; 1.89]	2.06 [1.78; 2.38]
Psychiatric visit	1.61 [1.56; 1.67]	1.43 [1.38; 1.48]	1.34 [1.23; 1.45]
Psychiatric hospitalisation	2.09 [1.99; 2.21]	1.77 [1.67; 1.87]	1.75 [1.54; 1.99]
Premature mortality	2.26 [1.95; 2.61]	1.94 [1.68; 2.24]	1.92 [1.34; 2.74]
Low education	1.69 [1.62; 1.75]	1.53 [1.48; 1.59]	1.37 [1.27; 1.49]
Welfare recipiency	1.64 [1.58; 1.70]	1.34 [1.29; 1.39]	1.21 [1.12; 1.32]

Notes: Model I: Full sample, adjusted for sex, birth order, and birth year. Model II: Additional adjustments for individual and parental highest achieved education levels, parental income, parental lifetime criminal and psychiatric histories, and maternal single status. Model III: Within-family estimates that are additionally adjusted for individual educational attainment at age 26 y.

Stratifying the TBI exposure across injury severity (Table 5), we found that the estimates of mild TBI exposure were only slightly attenuated compared to the TBI estimates. Importantly, we observed that moderate-to-severe TBI was a stronger risk factor for all functioning outcomes. This was particularly evident in the case of premature mortality, for which moderate-to-severe TBI patients had an approximately 2-fold increase in risk compared to both unrelated controls (RR = 2.26; 1.95–2.61) and their unaffected siblings (RR = 1.92; 1.34–2.74). We found similar results for disability pension and psychiatric inpatient hospitalisation.

The risks of poor functioning in adulthood were substantially elevated across all outcomes in individuals who had suffered recurrent TBIs compared to those who had suffered a single TBI (Table 6). Recurrent TBI was, for instance, associated with a nearly 3-fold increase in risk of being in receipt of disability pension (RR = 2.58; 2.41–2.76) compared to unrelated controls that had sustained a single TBI and a 2-fold increase in risk compared to their siblings that had sustained a single TBI (RR = 2.22; 1.88–2.63). Similarly, we observed that the risks of psychiatric inpatient hospitalisation (RR = 1.84; 1.73–1.95) and premature mortality (RR = 2.11; 1.78–2.49) were doubled in individuals with recurrent injuries compared to those who had suffered a single TBI. These increases in risk persisted in the sibling-comparison models for both psychiatric inpatient hospitalisation (RR = 1.53; 1.31–1.79) and premature mortality (RR = 1.59; 1.02–2.49). Further stratification of the recurrence effects across injury severity revealed that individuals who had sustained multiple moderate to severe TBIs had a 6-fold increase in risk of being in receipt of disability pension (RR = 5.84; 5.17–6.60) when compared to population controls who were exposed to a single moderate-to-severe TBI, which persisted when further compared with their siblings who were similarly exposed to a single moderate-to-severe TBI (RR = 6.13; 4.18–8.99).

**Table 6:** Relative risks (RR) and corresponding 95% confidence intervals (CIs) for the associations between recurrent TBI compared to single-episode TBI, across injury severity, before age 25 y and poor functioning in adulthood.

	Model I	Model II	Model III
	RR [95% CI]	RR [95% CI]	RR [95% CI]
**Any TBI**			
Disability pension	2.58 [2.41; 2.76]	2.16 [2.02; 2.31]	2.22 [1.88; 2.63]
Psychiatric visit	1.52 [1.46; 1.59]	1.36 [1.30; 1.42]	1.24 [1.12; 1.38]
Psychiatric hospitalisation	1.84 [1.73; 1.95]	1.59 [1.49; 1.69]	1.53 [1.31; 1.79]
Premature mortality	2.11 [1.78; 2.49]	1.83 [1.55; 2.17]	1.59 [1.02; 2.49]
Low education	1.55 [1.48; 1.63]	1.44 [1.37; 1.51]	1.28 [1.15; 1.43]
Welfare recipiency	1.49 [1.42; 1.56]	1.24 [1.19; 1.30]	1.13 [1.02; 1.25]
**Mild TBI**			
Disability pension	1.69 [1.54; 1.85]	1.43 [1.30; 1.57]	1.45 [1.17; 1.80]
Psychiatric visit	1.44 [1.37; 1.53]	1.30 [1.23; 1.38]	1.20 [1.06; 1.37]
Psychiatric hospitalisation	1.79 [1.66; 1.93]	1.57 [1.45; 1.69]	1.55 [1.28; 1.89]
Premature mortality	1.59 [1.26; 2.01]	1.40 [1.11; 1.77]	1.65 [0.90; 3.02]
Low education	1.44 [1.35; 1.54]	1.33 [1.24; 1.42]	1.13 [0.98; 1.30]
Welfare recipiency	1.50 [1.41; 1.59]	1.28 [1.21; 1.36]	1.12 [0.98; 1.27]
**Moderate to severe TBI**			
Disability pension	5.84 [5.17; 6.60]	5.10 [4.51; 5.76]	6.13 [4.18; 8.99]
Psychiatric visit	1.53 [1.38; 1.69]	1.38 [1.25; 1.53]	1.31 [1.02; 1.68]
Psychiatric hospitalisation	1.42 [1.22; 1.66]	1.24 [1.07; 1.45]	1.14 [0.78; 1.66]
Premature mortality	2.26 [1.61; 3.18]	1.96 [1.39; 2.76]	1.72 [0.60; 4.90]
Low education	1.48 [1.33; 1.64]	1.40 [1.25; 1.56]	1.21 [0.95; 1.54]
Welfare recipiency	1.26 [1.13; 1.41]	1.05 [0.94; 1.18]	1.01 [0.80; 1.26]

Notes: Model I: Full sample, adjusted for sex, birth order, and birth year. Model II: Additional adjustments for individual and parental highest achieved education levels, parental income, parental lifetime criminal and psychiatric histories, and maternal single status. Model III: Within-family estimates that are additionally adjusted for individual educational attainment at age 26 y.

The age at first TBI was a very strong moderator of poor functioning in adulthood (Table 7; Model III findings in S5 Table). We observed a positive association between older age at first TBI and all outcomes, and in particular among those who sustained their first injury after the age of 15 y. The largest observed differences were found for psychiatric inpatient hospitalisation, for which the first TBI exposure between ages 0–4 y was associated with a 1.2 fold (RR = 1.24; 1.15–1.34) increased risk, while the equivalent estimate for ages 20–24 y was a 2.5-fold risk increase (RR = 2.47; 2.37–2.58). In complementary sensitivity analyses, we replicated these findings for mild TBI (S6 Table), which suggests that these findings could not be attributed to increased injury severity among those who sustained their first injury at an older age.

**Table 7:** Relative risks (RR) and corresponding 95% confidence intervals (CIs) for the associations between TBI and poor functioning in adulthood, across age-band at first injury.

	**Ages 0–4 y**	**Ages 5–9 y**	**Ages 10–14 y**	**Ages 15–19 y**	**Ages 20–24 y**
	**RR [95% CI]**	**RR [95% CI]**	**RR [95% CI]**	**RR [95% CI]**	**RR [95% CI]**
Disability pension	1.39 [1.28; 1.51]	1.37 [1.28; 1.48]	1.58 [1.47; 1.69]	1.85 [1.75; 1.97]	1.97 [1.87; 2.08]
Psychiatric visit	1.18 [1.13; 1.24]	1.19 [1.14; 1.24]	1.40 [1.35; 1.46]	1.60 [1.55; 1.66]	1.78 [1.73; 1.84]
Psychiatric hospitalisation	1.24 [1.15; 1.34]	1.33 [1.25; 1.42]	1.68 [1.58; 1.78]	2.04 [1.94; 2.14]	2.47 [2.37; 2.58]
Premature mortality	1.28 [1.12; 1.46]	1.40 [1.25; 1.56]	1.45 [1.29; 1.63]	1.76 [1.59; 1.96]	2.25 [2.07; 2.44]
Low education	1.32 [1.26; 1.39]	1.24 [1.19; 1.30]	1.43 [1.37; 1.50]	1.73 [1.67; 1.79]	1.67 [1.62; 1.73]
Welfare recipiency	1.33 [1.27; 1.40]	1.33 [1.27; 1.38]	1.40 [1.34; 1.46]	1.56 [1.50; 1.61]	1.70 [1.65; 1.76]

## Discussion

In this population-based register study of over 1.1 million Swedish children, we examined the associations between TBI exposure, identified from inpatient and outpatient episodes, from birth up to age 25, and a range of adverse adult medical and social outcomes with public health implications. We followed the sample for up to 41 y, examined 683,860 siblings in addition to population controls, and the investigation was sufficiently powered to reliably explore the moderating effects of age categories at first injury. There were five principal findings. First, we found that being exposed to a mild TBI was associated with a range of adverse outcomes, including disability pension, psychiatric visits and inpatient hospitalisation, premature mortality, low educational attainment, and welfare recipiency. To provide more accurate estimates, we used unaffected siblings as comparisons, which demonstrated that although there was some familial confounding (shared genetic and childhood family environmental risks), we found that mild TBI was associated with 18%–52% increased risk of these outcomes. In particular, the risks were highest for psychiatric inpatient hospitalisation and disability pension.

Second, we found that greater injury severity was associated with poorer functioning outcomes. Compared to mild TBI, exposure to moderate-to-severe TBI increased the risk of disability pension by 106% and of psychiatric inpatient hospitalisation by 92% when compared to their unaffected siblings. Importantly, we observed smaller differences between the population and sibling-comparison estimates (Model I and III) in the case of moderate-to-severe TBI compared to mild TBI, which provides additional evidence for a potentially causal effect of more severe TBI on adverse outcomes.

The third main finding was that recurrent TBIs led to poorer outcomes than a single TBI. This was most noticeable for the likelihood of receiving disability pension, for which the risk increase between differentially exposed siblings was around 2-fold for recurrent TBIs compared to a single TBI. One in eleven (*n* = 1,139; 9.0%) individuals who sustained recurrent TBIs went on to receive a disability pension, which is 759 individuals more than we would have expected. This risk increase is associated with a substantial economic impact. The per person average cost of productivity loss for a single day of work absence in Sweden is estimated to be US$190 [39]. If we assume that these 759 individuals are absent from the labour market for a period of 30 y, this would roughly translate to a cumulative cost of US$1.3 million per person or US$987 million in total for productivity losses alone over the same time period. Generalising this to the US setting, with an annual 848,000 TBI patients under the age of 25 y [2], we would expect 34,600 of these individuals to be at risk for permanent labour market exclusion, equating to approximately US$1.1 trillion.

Fourth, we found that TBI accounted for between 2%–6% of the differences in the adverse outcomes following adjustments for familial confounders shared by differentially exposed siblings. These estimates are not insignificant. By way of comparison, the population attributable risk of severe mental illnesses on violent criminal convictions in Sweden has been estimated at 5% [40]. Importantly, as the examples presented above demonstrate, successful prevention efforts targeting high-risk individuals may have a notable impact on health care and other costs.

Fifth, we found that age at first head injury moderated the effects on long-term outcome. There was an age-related association in which older age at first head injury, and particularly after the age of 15 y, substantially increased the risks of all examined outcomes. In the case of premature mortality, we further minimised the risks of survival bias by initiating the follow-up period at the end of each exposure period. This approach thus allowed us to include individuals who had died before age 26. Although in need of replication, our findings are perhaps consistent with the hypothesis that neuroplasticity in early life may have long-term protective effects. These effects were most noticeable in relation to psychiatric hospitalisation for which the risk was substantially less among those who had sustained their first TBI between ages 0–4 y (RR = 1.24; 1.15–1.34) compared to those who had sustained their first injury between ages 20–24 y (RR = 2.47; 2.37–2.58). The neuroplasticity literature is currently mixed and inconclusive, largely due to similar methodological limitations as those outlined above [41–43]. Our robust findings, however, cannot be explained by differential injury levels, injury proneness across the examined age groups, survival bias, or familial selection factors. Importantly, our findings suggest that current clinical guidelines [3] should be revised to explicitly offer age-sensitive recommendations for follow-up interventions in childhood, adolescence, and early adulthood.

Two main findings—that TBI was associated with subsequent adverse adulthood outcomes, and the dose-response relationship with injury severity—are consistent with previous research [17–20,44], although these studies have mostly focused on small and selected samples that rely extensively on self-reported data either from (severely) injured patients or their caregivers; an approach subject to substantial bias [45]. Similar results have been reported in studies examining global disability indices [10], such as the extended Glasgow Outcome Scale (GOSE) [21]. Our third finding, on the effects of recurrent injuries, is broadly consistent with the emerging literature on the adverse consequences of sports and combat-related neurotrauma [7,9,11–14,46–49]. Although replication of this research is needed with larger samples of athletes and using research designs, such as comparisons with sibling controls, that account for confounding factors, our findings suggest that recurrent neurotrauma in any setting may be a causal risk factor for a wide range of social, cognitive, and medical adverse outcomes.

One of the implications of these findings is the importance of developing preventive interventions for early exposure to head injuries. In toddlers and preschoolers, these interventions should ideally be targeting improved parental supervision, as falling is the most common cause of TBI in this group. Prevention of sports-related concussions in older children could focus on changes to rules so that the risks of players colliding their heads with each other and with equipment (e.g., heading soccer balls or getting hit in the head by a racket, bat, or stick) [50] is reduced. A recent systematic review indicated that helmet use in most sports is generally associated with reduced risks for severe head injuries but not for concussions/mild TBI [51]. The development of safer helmets should also be prioritised as, for instance, recent evaluations of American football helmets report performance differences [52]. A further implication of the current study is that individuals who have suffered serious injuries should receive close follow-up interventions beyond medical care. This holds especially true if the injuries are recurrent or have occurred for the first time after the age of 20 y. The involvement of social services and work training opportunities may mitigate a potential downward social drift.

Our study has significant strengths that include (1) a very large sample size, enabling precise measures of associations between early TBI exposure and objectively defined indicators of medical and social functioning during adulthood to be defined; (2) the high quality of registers allowed selection bias to a minimum, as we were able to retain 86% of the targeted sample across 41 y of follow-up; and (3) the family-based research design allowed us to robustly test for the aggregated effects of all pre-injury and other selection characteristics shared within the family. Two limitations should be noted: We will have underestimated the prevalence of mild TBI. We used the National Patient Register, as adopted by previous studies [8,15,16], to ascertain TBI cases. This allowed comprehensive information on all individuals who sought medical treatment for their injuries, but will have underestimated milder forms of TBI, for example of the type prevalent in sports-related injuries [53–56]. This will not likely affect our main conclusions, as misclassifying head injured individuals as unaffected will have led to an underestimation of the presented associations, assuming that the effects of diagnosed and undiagnosed TBI on the outcomes are similar in magnitude. A co-twin design might better account for residual genetic confounds, but we were underpowered to do so, as it would require monozygotic twins that are discordant on head injuries, which would lead to considerably wider confidence intervals than traditional approaches [25].

The generalisability of our findings is an important consideration, and there is evidence that Sweden is similar to other high-income countries in terms of the key exposures and outcomes in this study. A recent systematic review [57] of annual TBI incidence rates for individuals up to age 20 y identified three studies from the US and New Zealand with a median estimate of 973 [range: 790–1,652] injuries per 100,000 population. We calculated equivalent estimates for Sweden between 2001 and 2009 and observed a median of 1,220 (range: 931–1,262) injuries per 100,000 population. Sweden’s employment rate (74.2%) was comparable to that of the United Kingdom (70.5%) and the US (67.3%) in 2013 [58]. The estimated life expectancy in Sweden is similar to that of other Organisation for Economic Co-operation and Development (OECD) countries [59], and a systematic review of the rates of mental disorders in Western European countries found no material country differences [60].

Children and adolescents who are exposed to TBI, even if the injuries appear to be relatively minor, have elevated risks to develop a wide range of medical and social problems during adulthood. The risks are substantially elevated as a function of injury severity, recurrence, and time of injury. These observations have potentially important implications for the way that paediatric TBI is currently clinically managed. Currently most children with significant head injuries receive no systematic long-term follow-up. An implication of our findings is that this should change. Services should consider how to routinely and systematically review these children on a regular basis to allow the subtle but important neurological, cognitive, and psychiatric consequences of TBI to be identified. Routinely tracking the progress of patients following significant TBI allows those with an altered developmental and educational trajectory to be identified and appropriate, age-sensitive interventions to be initiated. Detailed clinical studies on representative paediatric populations are needed to extend recent advances in the understanding of adult TBI pathophysiology, as this will inform the nature of key drivers for poor long-term outcome [61,62]. Effective prevention that minimises the risks of early exposure to TBI is required, and such efforts should benefit from large-scale epidemiological studies that identify potentially causal risk factors for the severity, recurrence, and developmental timing of TBI.

## Supporting Information

S1 STROBE Checklist STROBE statement. (DOCX)

S1 Table ICD diagnostic codes. (DOCX)

S2 Table Correlation table between the examined outcome variables (*n* = 1,143,470). (DOCX)

S3 Table Number of events, time at risk, and Kaplan-Meier estimate (KME) or prevalence rate of adulthood functioning outcomes across TBI exposure up to age 25 y in the full sample as well as the subsample of TBI-discordant siblings. (DOCX)

S4 Table Relative risks (RRs) and corresponding 95% confidence intervals (CIs) for the associations between TBI up to age 25 y and adulthood poor functioning in a subsample excluding any psychiatric or neurological conditions during the exposure period. (DOCX)

S5 Table Relative risks (RRs) and corresponding 95% confidence intervals (CIs) for the associations between TBI and adulthood poor functioning across age-band at first injury categories. (DOCX)

S6 Table Relative risks (RRs) and corresponding 95% confidence intervals (CIs) for the associations between mild TBI and adulthood poor functioning across age-band at first injury categories. (DOCX)

S1 Text Missing data analysis. (DOCX)

## Abbreviations

aRR: adjusted relative risk
ICD: International Classification of Diseases
PAF: population attributable fractions
RR: relative risk
RRECiS: Regional Research Ethics Committee in Stockholm
SEM: structural equation model
TBI: traumatic brain injury
TPR/MGR: Total Population/Multi-Generation Register
