# Supplementary material for: Long-Term Outcomes Associated with Traumatic Brain Injury in Childhood and Adolescence: A Nationwide Swedish Cohort Study of a Wide Range of Medical and Social Outcomes
Source: PLoS Med. 2016 Aug 23;13(8):e1002103. doi: 10.1371/journal.pmed.1002103 (PMC4995002; doi:10.1371/journal.pmed.1002103)
Supplement: S1 STROBE Checklist — (DOCX) [file pmed.1002103.s001.docx]

**S1 STROBE Checklist. STROBE statement**

|  | Item No | Recommendation | Section paragraph(s) |
| --- | --- | --- | --- |
| **Title and abstract** | 1 | (*a*) Indicate the study’s design with a commonly used term in the title or the abstract.  The study design is included in the title of the paper: "Long-term outcomes associated with traumatic brain injury in childhood and adolescence: A nationwide Swedish cohort study of a wide range of medical and social outcomes. | - |
|  |  | (b) Provide in the abstract an informative and balanced summary of what was done and what was found.  We have provided this information in the Methods and Findings sections of our abstract:  ”Methods  In a Swedish birth cohort between 1973 and 1985 of 1,143,470 individuals, we identified all those who had sustained at least one TBI (n=104,290 or 9.1%) up to age 25 years and their unaffected siblings (n=68,268) using patient registers. We subsequently followed these individuals up for the following outcomes using multiple national registries: disability pension, specialist diagnoses of psychiatric disorders and psychiatric inpatient hospitalisation, premature mortality (before age 41 years), low educational attainment (not having achieved secondary school qualifications) and receiving means-tested welfare benefits. We used logistic and Cox regression models to quantify the association between TBI and specified adverse outcomes on the individual level. We further estimated population attributable fractions (PAF) for each outcome measure. We also compared differentially exposed siblings to account for unobserved genetic and environmental confounding. In addition to relative risk estimates, we also examined absolute risks by calculating prevalence and Kaplan-Meier estimates. In complementary analyses, we tested whether the findings were moderated by injury severity, recurrence, and age at first injury (ages 0-4, 5-9, 6-10, 15-19 and 20-24 years).  Findings  TBI exposure was associated with elevated risks of impaired adult functioning across all outcome measures. After a median follow-up period of 8 years from age 26 years, we found that TBI contributed to absolute risks of over 10% for specialist diagnoses of psychiatric disorders, low educational attainment, approximately 5% for disability pension, and 2% for premature mortality. The highest relative risks, adjusted for sex, birth year and birth order, were found for psychiatric inpatient hospitalisation (adjusted relative risk (aRR)=2.0; 95% CI: 1.9-2.0; 6,632 vs. 37,095 events), disability pension (aRR=1.8; 95% CI: 1.7-1.8; 4,691 vs. 29,778 events) and premature mortality (aRR=1.7; 95% CI: 1.6-1.9; 799 vs. 4,695 events). These risks were only marginally attenuated when the comparisons were made with their unaffected siblings, which implies that the effects of TBI were consistent with a causal inference. A dose-response relationship was observed with injury severity. Injury recurrence was also associated with higher risks – in particular for disability pension, we found that recurrent TBI was associated with a three-fold risk increase (aRR=2.6; 95% CI: 2.4-2.8) compared to a single-episode TBI. Higher risks for all outcomes were observed for those who had sustained their first injury at an older age (ages 20-24 years) with more than 25% increase in relative risk across all outcomes compared to the youngest age group (ages 0-4 years). On the population level, TBI explained between 2-6% of the variance in the examined outcomes.  Using hospital data underestimates milder forms of TBI but such misclassification bias suggests that the reported estimates are likely conservative. The sibling-comparison design accounts for unmeasured familial confounders shared by siblings, including half of their genes. Thus, residual genetic confounding remains a possibility but will unlikely alter our main findings as associations were only marginally attenuated within families.” | 2-3 |
| Introduction | | |  |
| Background/rationale | 2 | Explain the scientific background and rationale for the investigation being reported | 1-2 |
| Objectives | 3 | State specific objectives, including any prespecified hypotheses | 3 |
| Methods | | |  |
| Study design | 4 | Present key elements of study design early in the paper | Paragraph 3 in the introduction. |
| Setting | 5 | Describe the setting, locations, and relevant dates, including periods of recruitment, exposure, follow-up, and data collection | 1-3 |
| Participants | 6 | (*a*) Give the eligibility criteria, and the sources and methods of selection of participants. Describe methods of follow-up | 4-7 |
|  |  | (*b*) For matched studies, give matching criteria and number of exposed and unexposed | N/A |
| Variables | 7 | Clearly define all outcomes, exposures, predictors, potential confounders, and effect modifiers. Give diagnostic criteria, if applicable | 4-7 |
| Data sources/ measurement | 8* | For each variable of interest, give sources of data and details of methods of assessment (measurement). Describe comparability of assessment methods if there is more than one group | 4-7 |
| Bias | 9 | Describe any efforts to address potential sources of bias | 3 (Text S1), 10-12 |
| Study size | 10 | Explain how the study size was arrived at | 9-10 |
| Quantitative variables | 11 | Explain how quantitative variables were handled in the analyses. If applicable, describe which groupings were chosen and why | 7 |
| Statistical methods | 12 | (*a*) Describe all statistical methods, including those used to control for confounding | 9-13 |
|  |  | (*b*) Describe any methods used to examine subgroups and interactions | 11-12 |
|  |  | (*c*) Explain how missing data were addressed | 3 (Text S1) |
|  |  | (*d*) If applicable, explain how loss to follow-up was addressed | 9 |
|  |  | (*e*) Describe any sensitivity analyses | 11-12 |
| Results | | |  |
| Participants | 13* | (a) Report numbers of individuals at each stage of study—eg numbers potentially eligible, examined for eligibility, confirmed eligible, included in the study, completing follow-up, and analysed | 1 and Methods, paragraphs 3 and 9 |
|  |  | (b) Give reasons for non-participation at each stage | Methods, paragraphs 3 and 9 |
|  |  | (c) Consider use of a flow diagram | - |
| Descriptive data | 14* | (a) Give characteristics of study participants (eg demographic, clinical, social) and information on exposures and potential confounders | 1 |
|  |  | (b) Indicate number of participants with missing data for each variable of interest | N/A |
|  |  | (c) Summarise follow-up time (eg, average and total amount) | Table S3 |
| Outcome data | 15* | Report numbers of outcome events or summary measures over time | Table S3 |
| Main results | 16 | (*a*) Give unadjusted estimates and, if applicable, confounder-adjusted estimates and their precision (eg, 95% confidence interval). Make clear which confounders were adjusted for and why they were included | 2 |
|  |  | (*b*) Report category boundaries when continuous variables were categorized | N/A |
|  |  | (*c*) If relevant, consider translating estimates of relative risk into absolute risk for a meaningful time period | Table S3 |
| Other analyses | 17 | Report other analyses done—eg analyses of subgroups and interactions, and sensitivity analyses | 3-7 (Tables S4-S6) |
| Discussion | | |  |
| Key results | 18 | Summarise key results with reference to study objectives | 1-5 |
| Limitations | 19 | Discuss limitations of the study, taking into account sources of potential bias or imprecision. Discuss both direction and magnitude of any potential bias | 8 |
| Interpretation | 20 | Give a cautious overall interpretation of results considering objectives, limitations, multiplicity of analyses, results from similar studies, and other relevant evidence | 7,10 |
| Generalisability | 21 | Discuss the generalisability (external validity) of the study results | 9 |
| Other information | | |  |
| Funding | 22 | Give the source of funding and the role of the funders for the present study and, if applicable, for the original study on which the present article is based.  We have included this information in our financial disclosure:  ”The study was supported by the Wellcome Trust (095806), the Swedish Council for Working Life and Social Research, the Swedish Research Council (2010-3184; 2011-2492; 2013-5867) and the National Institute of Child Health and Human Development (HD061817). DJS is supported by a National Institute for Health Research (NIHR) Professorship (NIHR-RP-011-048). HL has served as a speaker for Eli-Lilly and Shire and has received a research grant from Shire; all outside the submitted work. BMD is receiving funding from the American Foundation for Suicide Prevention and the Indiana Clinical and Translational Sciences Institute, both outside of the submitted work. The funders were not involved in the design and conduct of the study; collection, management, analysis, and interpretation of the data; or preparation, review, or approval of the manuscript.” | - |

*Give information separately for exposed and unexposed groups.

**Note:** Information on the STROBE Initiative is available at http://www.strobe-statement.org.
