# Supplementary material for: Long-Term Outcomes Associated with Traumatic Brain Injury in Childhood and Adolescence: A Nationwide Swedish Cohort Study of a Wide Range of Medical and Social Outcomes
Source: PLoS Med. 2016 Aug 23;13(8):e1002103. doi: 10.1371/journal.pmed.1002103 (PMC4995002; doi:10.1371/journal.pmed.1002103)
Supplement: S1 Table — (DOCX) [file pmed.1002103.s002.docx]

**S1 Table. ICD diagnostic codes**

|  | **ICD-8** | **ICD-9** | **ICD-10** |
| --- | --- | --- | --- |
| TBI | 800-804, 850–854 | 800-804, 850–854 | S01.0–S01.9, S02.0-S02.3, S02.7–S02.9, S04.0,  S06.0–S06.9, S07.0-S07.1, S07.8-S07.9, S09.7–S09.9, T01.0, T02.0, T04.0, T06.0, T90.1-T90.2, T90.4-T90.5, T90.8-T90.9 |
| Mild TBI  [excludes individuals who have sustained any moderate to severe TBI] | 850 | 850 | S06.0 |
| Moderate to severe TBI  [excl. individuals who have sustained any mild TBI] | TBI excl. 850 | TBI excl. 850 | TBI excl. S06.0 |
| Fall injuries  [excl. individuals who have sustained any TBI] | E880-E888 | E880-E888 | W00-W19 |
| Any psychiatric conditions | 290-315 | 290-319 | F00-F99 |
| Any neurological conditions | 320-358 | 320-359 | G00-G99 |
