# Supplementary material for: Long-Term Outcomes Associated with Traumatic Brain Injury in Childhood and Adolescence: A Nationwide Swedish Cohort Study of a Wide Range of Medical and Social Outcomes
Source: PLoS Med. 2016 Aug 23;13(8):e1002103. doi: 10.1371/journal.pmed.1002103 (PMC4995002; doi:10.1371/journal.pmed.1002103)
Supplement: S2 Table — (DOCX) [file pmed.1002103.s003.docx]

**S2 Table. Correlation table between the examined outcome variables (***n* = **1,143,470)**

|  | **Disability pension** | **Psychiatric visit** | **Psychiatric hospitalisation** | **Premature mortality** | **Low education** | **Welfare recipiency** |
| --- | --- | --- | --- | --- | --- | --- |
| **Disability pension** | 1.00 |  |  |  |  |  |
| **Psychiatric visit** | 0.66 | 1.00 |  |  |  |  |
| **Psychiatric hospitalisation** | 0.60 | 0.82 | 1.00 |  |  |  |
| **Premature mortality** | 0.38 | 0.34 | 0.47 | 1.00 |  |  |
| **Low education** | 0.47 | 0.36 | 0.38 | 0.26 | 1.00 |  |
| **Welfare recipiency** | 0.48 | 0.53 | 0.55 | 0.32 | 0.51 | 1.00 |

Notes: The correlations are estimated using the tetrachoric approach (tetrachoric command in Stata 14 MP), which assumes that all of the outcome measures are binary. All of the correlations are statistically significant (p<0.001).
