# Supplementary material for: Long-Term Outcomes Associated with Traumatic Brain Injury in Childhood and Adolescence: A Nationwide Swedish Cohort Study of a Wide Range of Medical and Social Outcomes
Source: PLoS Med. 2016 Aug 23;13(8):e1002103. doi: 10.1371/journal.pmed.1002103 (PMC4995002; doi:10.1371/journal.pmed.1002103)
Supplement: S3 Table — (DOCX) [file pmed.1002103.s004.docx]

**S3 Table. Number of events, time at risk, and Kaplan-Meier estimate (KME) or prevalence rate of adulthood functioning outcomes across TBI exposure up to age 25 y in the full sample as well as the subsample of TBI-discordant siblings.**

|  | **No TBI** | | | | | | **TBI** | | | | | |
| --- | --- | --- | --- | --- | --- | --- | --- | --- | --- | --- | --- | --- |
|  | **Full sample (n=1,039,180)** | | | **TBI discordant siblings (n=68,268)** | | | **Full sample (n=104,290)** | | | **TBI discordant siblings (n=55,831)** | | |
|  | **Number of cases** | **Time at risk: Total (mean)** | **KME or**  **prevalence rate [95% CI]** | **Number of cases** | **Time at risk: Total (mean)** | **KME or**  **prevalence rate [95% CI]** | **Number of cases** | **Time at risk: Total (mean)** | **KME or**  **prevalence rate [95% CI]** | **Number of cases** | **Time at risk: Total (mean)** | **KME or**  **prevalence rate [95% CI]** |
| Disability pension | 29,778 | 8,245,321  (7.9) | 3.9%  [3.8%; 3.9%] | 1,956 | 520,084  (7.6) | 3.8%  [3.5%-4.0%] | 4,691 | 730,653  (7.0) | 6.3%  [6.0%; 6.7%] | 2,205 | 388,155  (7.0) | 5.5%  [5.0%; 5.9%] |
| Psychiatric visit | 105,393 | 8,218,209  (7.9) | 14.2%  [14.1%; 14.3%] | 7,191 | 521,203  (7.6) | 15.0%  [14.7%; 15.4%] | 14,586 | 725,927  (7.0) | 20.0%  [19.8%; 20.5%] | 7,094 | 389,482  (7.0) | 18.4%  [17.9%; 18.8%] |
| Psychiatric hospitalisation | 37,095 | 8,731,172  (8.4) | 5.7% [5.6%; 5.8%] | 2,604 | 551,482  (8.1) | 6.2%  [5.9%; 6.6%] | 6,632 | 775,324  (7.4) | 10.4%  [10.0%; 10.8%] | 3,101 | 412,780  (7.4) | 9.0%  [8.5%; 9.5%] |
| Premature mortality | 4,695 | 9,909,144  (9.5) | 0.8%  [0.8%; 0.9%] | 301 | 629,251  (9.2) | 0.8%  [0.7%; 1.0%] | 799 | 906,594  (8.7) | 1.6%  [1.4%; 1.8%] | 374 | 480,742  (8.6) | 1.4%  [1.2%; 1.8%] |
| Low education | 92,783 | N/A | 8.9%  [8.9%; 9.0%] | 6,918 | N/A | 10.1%  [9.9%; 10.4%] | 14,494 | N/A | 13.9%  [13.7%; 14.1%] | 6,980 | N/A | 12.5%  [12.3%; 12.8%] |
| Welfare recipiency | 90,117 | 7,856,291  (7.6) | 11.5%  [11.4%; 11.5%] | 6,630 | 491,585  (7.2) | 12.6%  [12.1%; 13.1%] | 13,026 | 683,560  (6.6) | 19.1%  [18.7%; 19.4%] | 6,052 | 367,201  (6.6) | 14.7%  [14.1%; 15.4%] |

Notes: The calculations are based on yearly data for disability pension and welfare recipiency and daily data for the remaining outcomes. Low educational attainment was measured at the baseline of the follow-up. N/A = not applicable.
