# Supplementary material for: Long-Term Outcomes Associated with Traumatic Brain Injury in Childhood and Adolescence: A Nationwide Swedish Cohort Study of a Wide Range of Medical and Social Outcomes
Source: PLoS Med. 2016 Aug 23;13(8):e1002103. doi: 10.1371/journal.pmed.1002103 (PMC4995002; doi:10.1371/journal.pmed.1002103)
Supplement: S4 Table — (DOCX) [file pmed.1002103.s005.docx]

**S4 Table. Relative risks (RRs) and corresponding 95% confidence intervals (CIs) for the associations between TBI up to age 25 y and adulthood poor functioning in a subsample excluding any psychiatric or neurological conditions during the exposure period.**

|  | **Model I** | **Model II** | **Model III** |
| --- | --- | --- | --- |
|  | **RR [95% CI]** | **RR [95% CI]** | **RR [95% CI]** |
| Disability pension | 2.68 [2.59; 2.78] | 2.26 [2.18; 2.35] | 2.31 [2.10; 2.53] |
| Psychiatric visit | 1,80 [1.76; 1.83] | 1.63 [1.60; 1.66] | 1.55 [1.48; 1.62] |
| Psychiatric hospitalisation | 2.42 [2.35; 2.49] | 2.12 [2.06; 2.18] | 1.96 [1.82; 2.12] |
| Premature mortality | 1.94 [1.78; 2.10] | 1.71 [1.58; 1.87] | 1.71 [1.36; 2.14] |
| Low education | 1.70 [1.67; 1.74] | 1.55 [1,51; 1.58] | 1.34 [1.28; 1.40] |
| Welfare recipiency | 1.69 [1.66; 1.73] | 1.41 [1.39; 1.44] | 1.27 [1.21; 1.33] |

Notes: Model I: Full sample, adjusted for sex, birth order and birth year; Model II: Additional adjustments for individual and parental highest achieved education levels, parental income, parental lifetime criminal and psychiatric histories, and maternal single status; Model III: Within-family estimates that are additionally adjusted for individual educational attainment at age 26 years.
