# Supplementary material for: Long-Term Outcomes Associated with Traumatic Brain Injury in Childhood and Adolescence: A Nationwide Swedish Cohort Study of a Wide Range of Medical and Social Outcomes
Source: PLoS Med. 2016 Aug 23;13(8):e1002103. doi: 10.1371/journal.pmed.1002103 (PMC4995002; doi:10.1371/journal.pmed.1002103)
Supplement: S5 Table — (DOCX) [file pmed.1002103.s006.docx]

**S5 Table. Relative risks (RRs) and corresponding 95% confidence intervals (CIs) for the associations between TBI and adulthood poor functioning across age-band at first injury categories.**

|  | **Model I** | | | | | **Model III** | | | | |
| --- | --- | --- | --- | --- | --- | --- | --- | --- | --- | --- |
|  | **Ages 0-4 years** | **Ages 5-9 years** | **Ages 10-14 years** | **Ages 15-19 years** | **Ages 20-24 years** | **Ages 0-4 years** | **Ages 5-9 years** | **Ages 10-14 years** | **Ages 15-19 years** | **Ages 20-24 years** |
|  | **RR [95% CI]** | **RR [95% CI]** | **RR [95% CI]** | **RR [95% CI]** | **RR [95% CI]** | **RR [95% CI]** | **RR [95% CI]** | **RR [95% CI]** | **RR [95% CI]** | **RR [95% CI]** |
| Disability pension | 1.39 [1.28; 1.51] | 1.37 [1.28; 1.48] | 1.58 [1.47; 1.69] | 1.85 [1.75; 1.97] | 1.97 [1.87; 2.08] | 1.04 [0.86; 1.24] | 1.29 [1.10; 1.50] | 1.28 [1.09; 1.49] | 1.49 [1.30; 1.70] | 1.73 [1.53; 1.96] |
| Psychiatric visit | 1.18 [1.13; 1.24] | 1.19 [1.14; 1.24] | 1.40 [1.35; 1.46] | 1.60 [1.55; 1.66] | 1.78 [1.73; 1.84] | 1.02 [0.92; 1.13] | 1.11 [1.02; 1.22] | 1.28 [1.17; 1.40] | 1.24 [1.15; 1.34] | 1.53 [1.42; 1.64] |
| Psychiatric hospitalisation | 1.24 [1.15; 1.34] | 1.33 [1.25; 1.42] | 1.68 [1.58; 1.78] | 2.04 [1.94; 2.14] | 2.47 [2.37; 2.58] | 1.00 [0.85; 1.19] | 1.07 [0.94; 1.23] | 1.42 [1.24; 1.63] | 1.62 [1.43; 1.83] | 1.92 [1.73; 2.14] |
| Premature mortality | 1.28 [1.12; 1.46] | 1.40 [1.25; 1.56] | 1.45 [1.29; 1.63] | 1.76 [1.59; 1.96] | 2.25 [2.07; 2.44] | 1.28 [0.97; 1.68] | 1.14 [0.91; 1.44] | 1.26 [0.98; 1.62] | 1.24 [1.00; 1.55] | 1.59 [1.30; 1.59] |
| Low education | 1.32 [1.26; 1.39] | 1.24 [1.19; 1.30] | 1.43 [1.37; 1.50] | 1.73 [1.67; 1.79] | 1.67 [1.62; 1.73] | 1.00 [0.90; 1.11] | 1.10 [1.01; 1.21] | 1.22 [1.11; 1.34] | 1.41 [1.31; 1.53] | 1.34 [1.24; 1.44] |
| Welfare recipiency | 1.33 [1.27; 1.40] | 1.33 [1.27; 1.38] | 1.40 [1.34; 1.46] | 1.56 [1.50; 1.61] | 1.70 [1.65; 1.76] | 1.03 [0.93; 1.13] | 1.07 [0.98; 1.17] | 1.19 [1.09; 1.31] | 1.20 [1.11; 1.29] | 1.24 [1.16; 1.33] |

Notes: Model I: Full sample, adjusted for sex, birth order and birth year; Model III: Within-family estimates that are additionally adjusted for individual educational attainment at age 26 years. Adjustments for educational attainment are not made in the case of premature mortality to account for mortality cases occurring prior to age 26 years.
