# Supplementary material for: Long-Term Outcomes Associated with Traumatic Brain Injury in Childhood and Adolescence: A Nationwide Swedish Cohort Study of a Wide Range of Medical and Social Outcomes
Source: PLoS Med. 2016 Aug 23;13(8):e1002103. doi: 10.1371/journal.pmed.1002103 (PMC4995002; doi:10.1371/journal.pmed.1002103)
Supplement: S1 Text — (DOCX) [file pmed.1002103.s008.docx]

**S1 Text. Missing data analysis**

We examined the ways in which our findings were potentially biased by the relatively small share of missing individuals (n=190,449 or 14.3%) in three separate steps. First, we tested whether there were any gender and/or birth year differences across the included and the missing sub-samples. We found statistically significant (p<0.001) but clinically negligible differences for both gender (48.3% vs. 50.7% females) and birth year (1978.8 vs. 1978.7).

Second, we explored whether the rates (per 1,000 person-years) of TBI differed between the mentioned sub-samples. We found that the rate of TBI in the included sub-sample was higher (5.31; 95% CI: 5.28-5.33) than that of the missing sub-sample (3.04; 95% CI: 2.98-3.10). However, we were only able to follow the missing cases up to an average age of 16.2 years, which implies that they did not have enough time to be exposed to TBI during the high-risk period of late adolescence and early adulthood.

Third, we examined the associations between TBI and the adverse outcomes in 54,463 individuals who had data on the exposure and the outcomes but who were excluded from the main analyses due to lacking parental data. We found that the relative risks in this sub-sample were considerably elevated when compared to those who were included in the main analyses.

|  | **Main sample** | **Excluded sample** |
| --- | --- | --- |
|  | **RR [95% CI]** | **RR [95% CI]** |
| Disability pension | 1.76 [1.71; 1.82] | 2.45 [2.26; 2.65] |
| Psychiatric visit | 1.53 [1.50; 1.55] | 2.70 [2.47; 2.95] |
| Psychiatric hospitalisation | 1.95 [1.90; 2.00] | 3.40 [3.01; 3.84] |
| Premature mortality | 1.73 [1.60; 1.86] | 3.33 [2.70; 4.11] |
| Low educational attainment | 1.58 [1.55; 1.61] | 2.15 [1.87; 2.48] |
| Welfare recipiency | 1.55 [1.52; 1.58] | 3.01 [2.71; 3.35] |

Note: All models have been adjusted for gender and birth year.

Together, these results suggest that our presented estimates are likely to be conservative.
